# Unveiling the transcriptomic complexity of *Miscanthus sinensis* using a combination of PacBio long read- and Illumina short read sequencing platforms

**DOI:** 10.1186/s12864-021-07971-x

**Published:** 2021-09-22

**Authors:** Yongli Wang, Xia Li, Congsheng Wang, Lu Gao, Yanfang Wu, Xingnan Ni, Jianzhong Sun, Jianxiong Jiang

**Affiliations:** Biofuels Institute, School of the Environment and Safety Engineering, Jiangsu University, 212013 Zhenjiang, Jiangsu China

**Keywords:** *Miscanthus sinensis*, PacBio Iso-Seq, Illumina HiSeq, Transcriptomic analysis, Transcription factors, Alternative splicing

## Abstract

**Background:**

*Miscanthus sinensis* Andersson is a perennial grass that exhibits remarkable lignocellulose characteristics suitable for sustainable bioenergy production. However, knowledge of the genetic resources of this species is relatively limited, which considerably hampers further work on its biology and genetic improvement.

**Results:**

In this study, through analyzing the transcriptome of mixed samples of leaves and stems using the latest PacBio Iso-Seq sequencing technology combined with Illumina HiSeq, we report the first full-length transcriptome dataset of *M. sinensis* with a total of 58.21 Gb clean data. An average of 15.75 Gb clean reads of each sample were obtained from the PacBio Iso-Seq system, which doubled the data size (6.68 Gb) obtained from the Illumina HiSeq platform. The integrated analyses of PacBio- and Illumina-based transcriptomic data uncovered 408,801 non-redundant transcripts with an average length of 1,685 bp. Of those, 189,406 transcripts were commonly identified by both methods, 169,149 transcripts with an average length of 619 bp were uniquely identified by Illumina HiSeq, and 51,246 transcripts with an average length of 2,535 bp were uniquely identified by PacBio Iso-Seq. Approximately 96 % of the final combined transcripts were mapped back to the *Miscanthus* genome, reflecting the high quality and coverage of our sequencing results. When comparing our data with genomes of four species of Andropogoneae, *M. sinensis* showed the closest relationship with sugarcane with up to 93 % mapping ratios, followed by sorghum with up to 80 % mapping ratios, indicating a high conservation of orthologs in these three genomes. Furthermore, 306,228 transcripts were successfully annotated against public databases including cell wall related genes and transcript factor families, thus providing many new insights into gene functions. The PacBio Iso-Seq data also helped identify 3,898 alternative splicing events and 2,963 annotated AS isoforms within 10 function categories.

**Conclusions:**

Taken together, the present study provides a rich data set of full-length transcripts that greatly enriches our understanding of *M. sinensis* transcriptomic resources, thus facilitating further genetic improvement and molecular studies of the *Miscanthus* species.

**Supplementary Information:**

The online version contains supplementary material available at 10.1186/s12864-021-07971-x.

## Background

*Miscanthus sinensis* Andersson (Poaceae) is considered to be one of the most promising lignocellulosic bioenergy crops owing to its high biomass yield, ability to grow on marginal land, and low fertilizer requirements [1]. However, as a non-model species, dedicated functional genomics and transcriptomic resources for *Miscanthus* are still poorly explored owing to the high heterozygosity and complex ploidy of their genomes, which becomes a bottleneck for understanding the molecular processes underlying its superior bioenergetic qualities. The best-known species of *Miscanthus* is *Miscanthus* × *giganteus*, which is a hybrid of *Miscanthus sinensis* and *Miscanthus sacchariflorus* (Maxim.) Franch, and has been commercially used as a biomass feedstock in Europe and North America [2]. However, owing to its sterility and narrow genetic basis, it is difficult to improve *M.* × *giganteus* through breeding. As a progenitor of *M.* × *giganteus*, *M. sinensis* is widely distributed throughout East Asia, especially in China where it provides yields comparable to *M.* × *giganteus* and exhibits good tolerance to stress [3]. Thus, understanding molecular processes underlying the basic biology of *M. sinensis* would enable agronomists to develop crops ideal for biomass production.

Owing to rapid developments in sequencing technologies, the availability of whole-genomic data has enjoyed exponential growth over the past years. The genomes of more than two hundred plant species have been completely sequenced; most of them are plants of high economic importance [4]. These data facilitate both rapid gene discovery and annotation of gene networks. However, only a part of the plant genomes have been well-constructed and utilized, and genomes of many other important crops still remain unknown due to the large size and complexity of their genomes. Transcriptome analysis using next-generation sequencing (NGS) represent a cost-effective method to explore diverse biological processes in non-model organisms [5–7]. For example, extensive transcriptome analyses have been conducted on switchgrass (*Panicum virgatum* L.), which is another important bioenergy crop widely used for lignocellulosic research. Comparative transcriptome analyses of different switchgrass ecotypes identified many molecular biomarkers of phenotypic traits, which accelerated the development of high-yielding switchgrass cultivars [8, 9]. Although several *Miscanthus* species have been considered important feedstocks for biofuels and biorefineries, their genetic resources, including transcriptome data, are currently very limited. Previously, transcriptome profiling has only been reported for three important *Miscanthus* species, including *M. sinensis*, *M. sacchariflorus*, and *M.* × *giganteus*, to study their transcriptomic similarity with other grasses, construct genetic maps using NGS based markers, and identify genes probably related to traits like rhizome development and self-incompatibility traits [10–12]. *Miscanthus sinensis*, a genetic diploid (2n = 38), has a genome size of one copy of a single complete genome of 2.4–2.6 Gb [13]. The key limitation to the genetic improvement of *Miscanthus sinensis* is the complexity of its genome, which makes transcriptome studies of *Miscanthus* very challenging. The NGS sequencing platform used in these studies offers a read-length of 25 to 400 bp, mostly between 100 and 200 bp, which is shorter than the typical length of eukaryotic mRNA (usually 1–3 kb, including a methylated cap at the 5’ end and poly-A at the 3’ end). In this case, low-quality transcripts derived from the mis-assembly of genes cannot be eliminated, which increased both the false positives (miss-predicted genes) and false negatives (over-looked real genes).

In recent years, next-generation short-read sequencing has been used to produce sequence data for many plant species, but knowledge of full-length (FL) sequences of mRNAs remains scarce. Considering the complexity of the transcriptome, it is very challenging to precisely predict and identify alternative transcript splicing using short-read data. Since the assemblers cannot distinguish between reads originally from different transcripts carrying the same exons, the identification of alternative splicing (AS) contributes significantly to enhance transcriptome diversity and assess a splice variant’s role in gene regulation [14]. As such, FL transcripts can significantly extend our understanding of the transcriptome and can increase the accuracy of genome annotation. With the rise of long read sequencing technologies, the length of sequencing reads has been dramatically increased. The PacBio Sequel™ system (PacBio, Menlo Park, CA, USA) can produce an average read length of more than 10 kb. This protocol is also called Isoform Sequencing (hereafter referred to as Iso-Seq), because by producing longer reads, the PacBio Iso-Seq technology eliminates the need for assembly, providing direct evidence for transcript isoforms of each gene [15]. This considerable advantage of Iso-Seq technology expands its utility to defining alternatively spliced forms and some non-coding RNAs that can vary with cell-type, developmental stage, or stress [16, 17]. The ability of Iso-Seq to produce vast Gb of relatively long sequence reads thus hold promising applications for crop genomics.

Thus far, the applications of Iso-Seq in plant science have been comparatively narrow, mostly limited to studying crops and/or model plant systems with well-constructed genome references. This is because the widely used Iso-Seq data analysis pipeline TAPIS (transcriptome analysis pipeline for isoform sequencing) relies on high-quality genome assembly and annotation to define isoforms, AS, and polyadenylation sites. For example, Iso-Seq with TAPIS has been recently applied in transcriptomic studies of maize and sorghum to identify full-length transcript isoforms and AS [18, 19]. The survey of transcriptome isoform diversity by using Iso-Seq is becoming a landmark for gene discovery and annotation in maize [18], sorghum [19], rice [20], safflower [21], and switchgrass [22]. However, the easy-to-use TAPIS reference-based pipeline cannot be applied to Iso-Seq data for error correction and *de novo* AS detection in species that lack a reference genome sequence. Recently, Liu et al. [23] described a pipeline for the detection of AS isoforms from PacBio Iso-Seq without using a reference sequence. Results using this pipeline showed a 66–76 % overall success rate in identifying AS events. The development of *de novo* AS detection pipelines promoted the applications of Iso-Seq in reconstructing a FL transcriptome in species without a reference genome. Thereby, Iso-Seq can provide a reference transcriptome for non-model plants whose reference genomes are not yet available, thus helping to produce more accurate gene models and contributing to the genetic improvement of these species. However, technical limitations such as high error rates and high costs make third-generation sequencing technologies unsuitable for immediate widespread applications [16]. Therefore, it is advantageous to combine Iso-Seq with NGS to generate more complete/full-length transcriptomic data. Many hybrid sequencing strategies have been developed to make use of both short reads and PacBio long reads [24, 25].

In this study, the latest PacBio Sequel platform was used to sequence the full-length transcriptome of *Miscanthus sinensis*. Illumina HiSeq producing high coverage short-read data was used in parallel to improve the PacBio transcript isoforms. Transcripts obtained or assembled from the two different platforms were compared and then merged into high-quality full-length transcripts. These transcripts were functionally annotated by integrating multiple bioinformatic databases including the Gene Ontology (GO) and the Kyoto encyclopedia of genes and genome (KEGG). Based on the high-accuracy of Iso-Seq data set, we detected AS events and full-length splice isoforms in *M. sinensis* by the *de novo* AS detection pipeline. Our results demonstrate the reliability and utility of PacBio Iso-Seq in characterizing transcripts and identifying novel genes/isoforms, particularly for species without a reference genome, which will not only improve genome annotation and enhance our understanding of the *Miscanthus* transcriptome, but will also expand our knowledge of the molecular basis of the plant’s biological processes.

## Results

### PacBio Iso-Seq sequencing of *Miscanthus* transcriptome

**Fig. 1:**
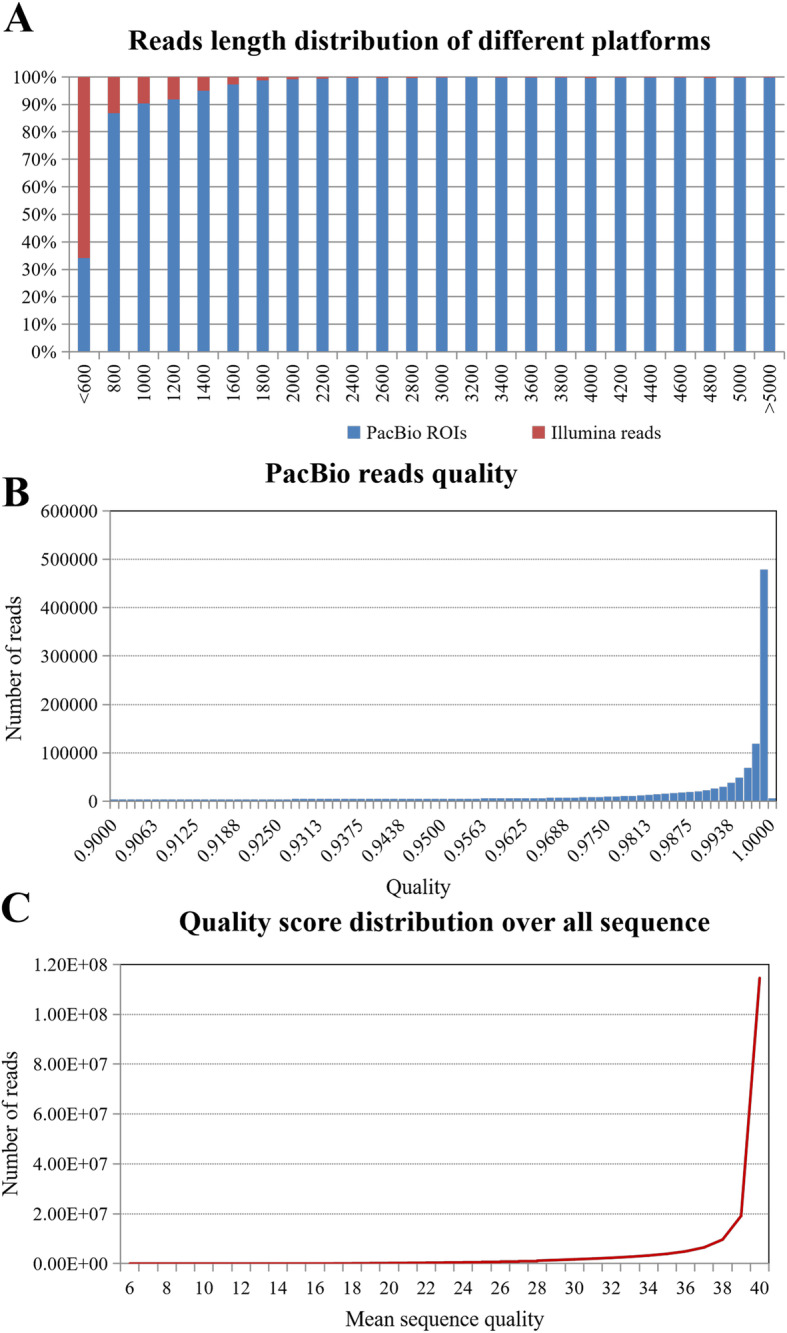
Length distribution and quality assessment of *Miscanthus sinensis* transcriptome datasets from two different sequencing platforms. **a** Comparison of read length distribution from PacBio Iso-Seq and Illumina HiSeq. **b** Sequencing quality of PacBio Iso-Seq data. **c** Sequencing quality of Illumina HiSeq data

**Fig. 2:**
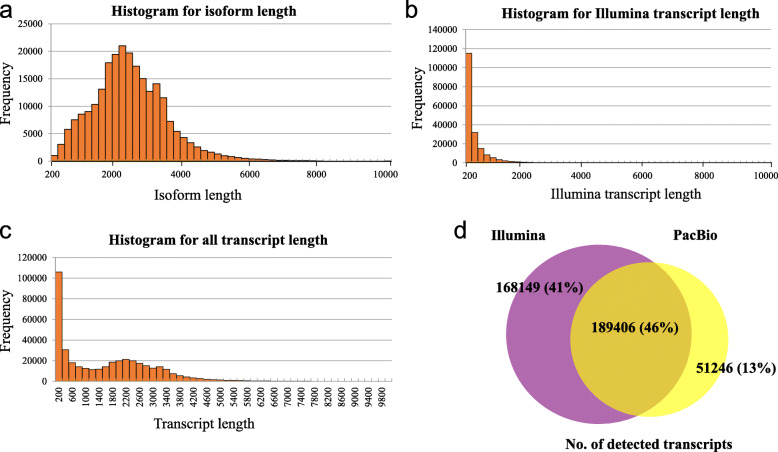
Summary of overall *Miscanthus sinensis* transcriptome transcripts by the combined analysis of PacBio Iso-Seq and Illumina HiSeq datasets. **a** Length distribution of Iso-Seq isoforms after cluster using CD-HIT-EST. **b** Length distribution of complemental transcripts assembled from Illumina HiSeq data. **c** Length distribution of overall *Miscanthus sinensis* transcriptome transcripts. **d** Venn diagram showing the common and unique transcripts detected by each method

To obtain a wide coverage of the *Miscanthus* transcriptome, high-quality mRNAs from leaves and stems of two *Miscanthus sinensis* genotypes (B0605 and C0542) were sequenced using the PacBio Iso-Seq Sequel platform. A total of 1,453,866,187 nucleotides were generated from the B0605 sample with a total of 647,252 multipass reads of inserts (ROIs), which included 597,037 (92.24 %) FL non-chimeric ROIs and 39,686 (6.13 %) non-FL ROIs (Table 1). The length of B0605 ROIs ranged from 200 bp to 15,600 bp, with a mean read length of 2,246 bp (Fig. 1a; Table 1). A total of 1,413,954,821 nucleotides were generated from the C0542 sample with a total of 635,350 ROIs, which included 583,088 (91.77 %) FL non-chimeric ROIs and 43,779 (6.89 %) non-FL ROIs (Table 1). The length of C0542 ROIs ranged from 200 bp to 14,000 bp, with a mean read length of 2,225 bp (Fig. 1a; Table 1). Overall, our PacBio Iso-Seq dataset consisted mostly of high-quality ROIs with quality values above 0.95, which is much higher than the quality of most PacBio ROIs reported in previous studies (above 0.85) (Fig. 1b) [18, 22]. Furthermore, we used an isoform-level clustering tool CD-HIT-EST to generate the cluster consensus of all the FLNC ROI sequences. In total, 240,665 final consensus isoforms were obtained with lengths ranging from 200 bp to 18,237 bp and a N50 value of 2,863 bp (Fig. 2a; Table 1).

**Table 1:** PacBio Iso-Seq output statistics

Samples		B0605	C0542
Summary of ROIs	Reads of Insert	647,252	635,350
	Read bases of insert (bp)	1,453,866,187	1,413,954,821
	Mean Read length of Insert (bp)	2,246	2,225
	Mean Read Quality of Insert	0.98	0.98
	Mean Number of Passes	11.31	10.70
Classification of ROIs	5’ primer reads	629,069	613,881
	3’ primer reads	630,499	616,089
	Poly-A reads	627,416	614,650
	Filtered reads	15	17
	NFL reads/percentage (%)	39,686	43,779
	FL reads/percentage (%)	607,551	591,554
	FLNC reads/percentage (%)	597,037	583,088
	Average FLNC read length (bp)	2,100	2,087
CD-HIT-EST cluster of isoforms	Number of consensus isoforms	240,665	
	Mean length of consensus isoforms (bp)	2,535	
	N50 (bp)	2,863	
	N90 (bp)	1,710

### Combined *Miscanthus* transcriptome with Illumina short-read sequencing

To provide high coverage sequence information, high-quality mRNAs from two biological replicates of the same tissues used for PacBio sequencing were simultaneously sequenced on the Illumina HiSeq 2,500 platform with pair-end of 150 bp. After cleaning and quality checks, more than 6 Gb of sequencing data with over 400 million total clean reads were obtained from each sample (Table 2). The Q20 % of each dataset was above 97.5 %, Q30 % of each dataset was above 94 % and quality scores of most reads exceeded 39 (Table 2; Fig. 1c). Overall, the length of Illumina reads was distributed among lower reads than PacBio reads and constituted 65.83 % of reads < 600 bp (Fig. 1a). The filtered Illumina read sequences of each sample were then aligned against the PacBio isoforms of the corresponding sample using Bowtie 2 [26] with the default highly sensitive settings. More than 90 % of Illumina reads were completely mapped to the PacBio isoforms of each sample, which revealed a good agreement between the short-read datasets and the long-read datasets at the nucleotide level (Table 2). The other clean reads that could not be mapped to the PacBio isoforms were further *de novo* assembled into 196,798 transcripts using the Trinity program [27]. The size of the assembled transcripts ranged from 200 to 8,526 bp, with a N50 value of 619 bp (Table 3; Fig. 2b). Among these transcripts, 147,335 (78.75 %) were shorter than 600 bp and only 16,206 (8.66 %) were longer than 1,000 bp (Fig. 2b).

**Table 2:** Illumina HiSeq output statistics and mapping results with PacBio Iso-Seq isoforms

Samples	Clean Data (bp)	Q20/Q30 %	N percentage	Clean reads No.	Total mapped
B0605-1	6,984,550,054	97.62 %/94.09 %	0.0018	47,049,590	43,350,530 (92.14 %)
B0605-2	6,291,808,850	97.68 %/94.21 %	0.0010	42,361,288	39,222,916 (92.59 %)
C0542-1	6,329,620,030	97.64 %/94.12 %	0.0014	42,617,610	38,886,988 (91.25 %)
C0542-2	6,580,610,956	97.59 %/94 %	0.0013	44,244,718	41,382,118 (93.53 %)

Q20**/**Q30 % represents the percentage of bases whose quality was larger than 20/30 in clean reads

**Table 3:** Summary of *Miscanthus sinensis* transcriptome transcripts

Sample	Total transcripts	Total bases (bp)	Max length (bp)	Mean length (bp)	N50 (bp)
PacBio	240,665	610,177,595	18,237	2,535	2,863
Illumina	196,798	99,323,094	8,526	505	619
Total	408,801	688,710,450	18,237	1,685	2,697

Furthermore, we used CD-HIT-EST to generate the cluster consensus of all the PacBio isoforms and Illumina assembled transcripts. A total of 408,801 final non-redundant transcripts were obtained after combining the two datasets with the length distributed from 200 bp to 18,237 bp and a N50 of value 2,697 bp (Table 3; Fig. 2c). Of all the detected transcripts, 189,406 (46 %) genes could be identified by both Illumina HiSeq and PacBio Iso-Seq platforms (Fig. 2d). Illumina HiSeq detected more uniquely identified genes (168,149, 41 %) than PacBio Iso-Seq (51,246, 13 %) (Fig. 2d), suggesting that the sequencing depth of PacBio Iso-Seq is lower than that of Illumina HiSeq. Moreover, to assess the completeness of conserved content of the 408,801 final non-redundant transcripts, BUSCO (Benchmarking Universal Single Copy Orthologs) was utilized to determine the completeness of our transcript dataset with the Viridiplantae (green plants) dataset [28]. The results showed that this dataset contains 87.5 % complete plus 11.5 % partial BUSCO orthologues (Additional file 1). Only a minority (0.9 %, 4 genes) of the genes was missing in our *Miscanthus* transcriptome, which indicated good coverage and transcriptome completion of the dataset.

### Comparison of the *Miscanthus* transcriptome with genomes of related species

As a non-model species in the Andropogoneae tribe, a draft chromosome-scale genome sequence for *M. sinensis* has recently been released, which is insufficient compared to other Andropogoneae crops with high quality, well annotated genomes such as rice, maize, sorghum, and sugarcane [29, 30]. The transcriptome is the comprehensive, functional readout of the genome. The availability of complete genome sequences of rice, maize, sorghum, sugarcane and the recently released miscanthus genome makes it possible to conduct comparative genomic analyses to gain a better understanding of the *Miscanthus* transcriptome [29–35]. In our study, the six sequenced datasets including two sets of PacBio FLNC ROIs and four sets of Illumina clean reads as well as all the final clustered transcripts were mapped to the genomes of rice [31], maize [32], sorghum [33], sugarcane [34] and *Miscanthus* [29] respectively (Table 4). For all the species, mapping ratios were highest with PacBio FLNC ROIs (34.92–99.80 %), followed by clustered transcripts (10.06–96.64 %), and then Illumina clean reads (0.6–87.02 %). This indicates that the Illumina-derived reads and *de novo* assembled transcripts may generate more sequence divergence than the PacBio Iso-Seq long isoforms. In total, 99 % of the PacBio reads, about 85 % of the Illumina reads, and 96 % of the final combined transcripts could be mapped back to the *Miscanthus* genome. The high mapping ratios between the *Miscanthus* transcriptome and genome reflects the high accuracy of PacBio sequencing and strengthened the confidence in our integrated approach.

**Table 4:** High-confidence mappings with genomes of *Miscanthus sinensis* and other four closely related plant species

Data type	*Oryza sativa*	*Zea mays*	*Sorghum bicolor*	*Saccharum Spontaneum L*	*Miscanthus sinensis*
PacBio B0605 FLNCs	210,330 (35.23 %)	463,741 (77.67 %)	478,942 (80.22 %)	557,818 (93.43 %)	593,760 (99.45 %)
PacBio C0542 FLNCs	203,587 (34.92 %)	450,169 (77.20 %)	464,189 (79.61 %)	545,601 (93.57 %)	581,923 (99.80 %)
Illumina B0605-1 Clean reads	330,754 (0.7 %)	6380,120 (13.5 %)	14,542,559 (30.7 %)	21,550,628 (45.6 %)	40,558,657 (85.82 %)
Illumina B0605-2 Clean reads	269,976 (0.6 %)	5,476,174 (12.9 %)	13,253,089 (31.1 %)	19,582,421 (46.0 %)	36,776,636 (86.39 %)
Illumina C0542-1 Clean reads	331,302 (0.8 %)	5,896,040 (13.8 %)	13,689,017 (31.9 %)	20,256,499 (47.3 %)	37,048,408 (86.51 %)
Illumina C0542-2 Clean reads	313,718 (0.7 %)	5,466,749 (12.3 %)	14,044,656 (31.6 %)	20,917,089 (47.0 %)	38,727,765 (87.02 %)
All transcripts	41,125 (10.06 %)	196,184 (47.99 %)	255,460 (62.49 %)	325,896 (79.72 %)	395,065 (96.64 %)

Values in brackets shows mapping ratios between two datasets

On the other hand, the mapping ratios of both the PacBio and Illumina-derived *Miscanthus* transcriptome sequences to the genome sequences of the four other species ranged from low to high, in the following order: maize, sorghum, sugarcane, and rice. The mapping ratios were the lowest when compared with the rice genome (0.6–35.23 %), which is unsurprising because rice is a C3 photosynthesis species while *Miscanthus* and the other three species are C4 photosynthesis species.

*Miscanthus* and the other three species that belong to the Saccharastrae subtribe were more closely related. Among them, the highest mapping ratios were identified between the *Miscanthus* and the sugarcane genome (45.6–93.57 %), followed by the sorghum genome (30.7–80.22 %), and the maize genome (12.3–77.67 %). These results provided new evidence to support previous phylogenetic studies of the relationships between Saccharastrae (which includes *Miscanthus*, maize, sorghum and sugarcane), *Saccharum* (including *Miscanthus*, sorghum and sugarcane), and the still unresolved interspecific breeding group “*Saccharum* complex” (which includes *Miscanthus* and sugarcane) [36].

### Transcript annotation and functional classification

**Fig. 3:**
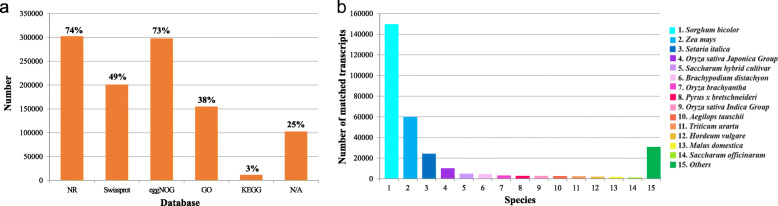
Function annotation of *Miscanthus sinensis* transcripts. **a** Function annotation of transcripts in all databases, NR, non-redundant protein database; Swiss-Prot, a manually annotated and reviewed protein sequence database; GO, Gene Ontology; COG/KOG, cluster of orthologous groups of proteins; KEGG, Kyoto Encyclopedia of Genes and Genomes; N/A, not available. **b** NR homologous species distribution diagram of transcripts

To confirm the putative function of the assembled transcripts, all 408,801 non-redundant transcripts were annotated using BLAST based on sequence similarity searches against five public protein databases, including the NR (NCBI non-redundant protein sequences), Swiss-Prot (a manually annotated and reviewed protein sequence database), eggNOG (evolutionary genealogy of genes: Non-supervised Orthologous Groups), GO (Gene Ontology), and KEGG (Kyoto Encyclopedia of Genes and Genome) databases, with an E-value threshold of 10^− 5^ (Additional file 2). In total, 306,228 (∼75 %) transcripts were successfully annotated in at least one of these five databases. Amongst them, 302,375 (∼74 %) transcripts had significant hits in Nr database, 201,067 (∼49 %) in Swiss-Prot database, 297,975 (∼73 %) in eggNOG database, 154,759 (∼38 %) in GO database, and 11,405 (∼3 %) in KEGG database (Fig. 3a). Meanwhile, 102,573 (∼25 %) transcripts remained unknown, so they can be considered as new transcripts putatively unique to *Miscanthus* (Additional file 2). Of all the hits to the NR proteins from BLASTX, most transcripts (149,618, ∼49 %) were annotated to *Sorghum bicolor*, followed by *Zea mays* (59,871; 19.8 %) (Fig. 3b), which is consistent with several previous *Miscanthus* transcriptome analyses when the sugarcane genome sequences were not yet available [10–12]. Thus, it is no surprise that most of our *Miscanthus* sequences could be mapped to the sugarcane genome but could not be annotated owing to the limited sugarcane genome annotation information in public databases.

**Fig. 4:**
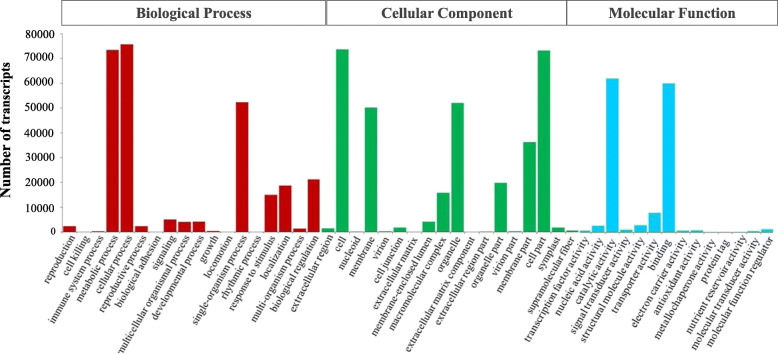
Histogram presentation of GO classification. Bars represent the numbers of transcripts distributed to 50 GO functional groups belonging to three categories: Biological process (red), Cellular component (green), and Molecular function (blue)

**Fig. 5:**
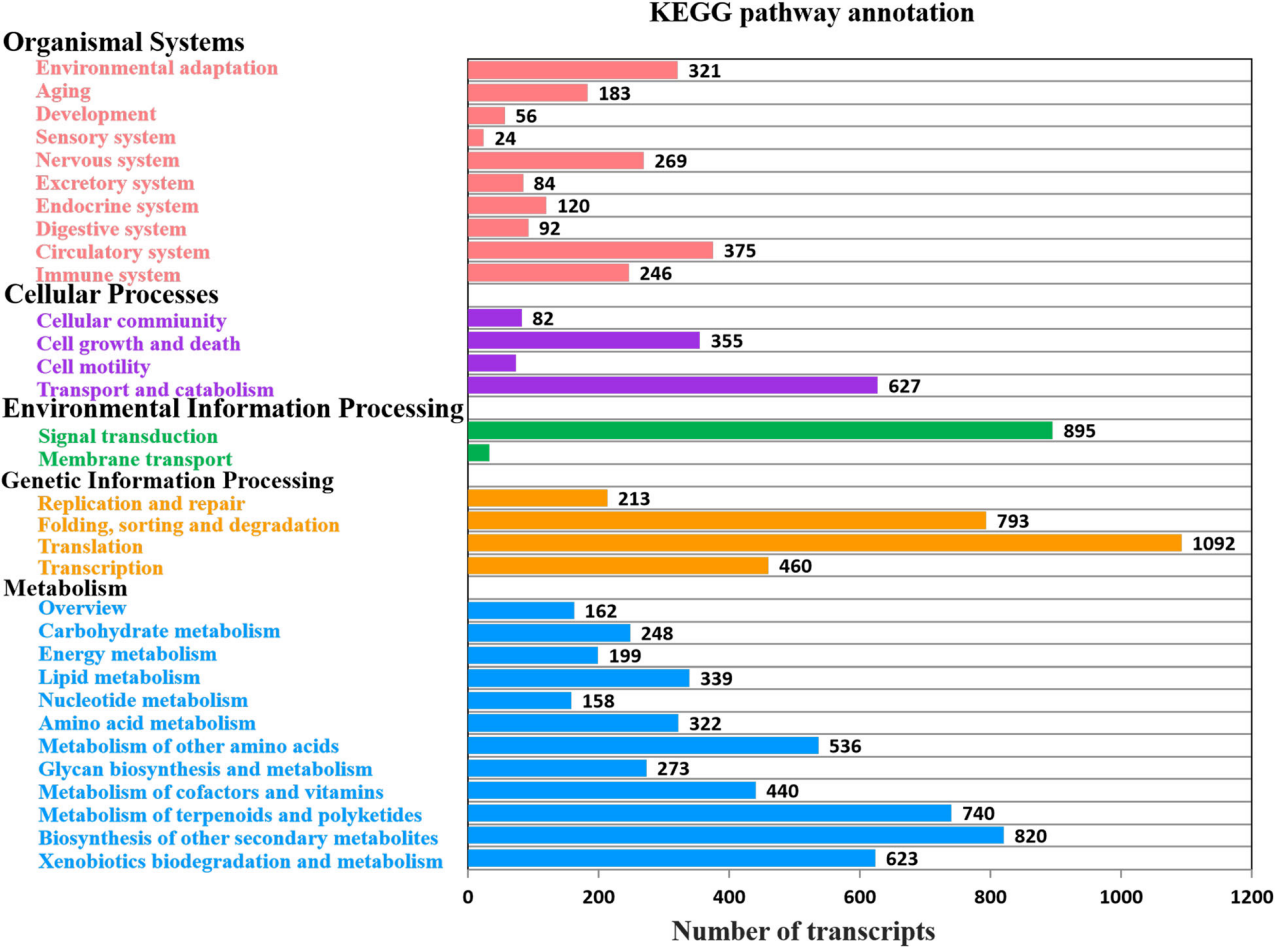
Histogram presentation of KEGG pathway classification. Bars represent the numbers of transcripts distributed to 32 KEGG pathway levels of five categories: Organismal systems (pink), Cellular processes (purple), Environmental information processing (green), Genetic information processing (orange) and Metabolism (blue)

As an international standardized gene functional classification system, GO offers a dynamic updated controlled vocabulary and a strictly defined concept to describe the properties of genes and their products in any organism [37, 38]. A total of 154,759 transcripts were annotated to at least one GO term, which were classified into 50 functional groups under three major functional categories (biological process, cellular component, and molecular function) (Fig. 4). In the category of biological process, the main groups were “cellular process” (75,733; 48.9 %), “metabolic process” (73,453; 47.5 %), and “single-organism process” (52,302, 33.8 %). In the cellular component category, transcripts related to the “cell” (72,611; 46.9 %), “cell part” (72,224; 46.7 %), and “organelle” (19,514; 12.6 %) were the best-represented groups. For the molecular function category, the most abundant transcripts were associated with “catalytic activity” (74,227; 48 %) and “binding” (71,790; 46.4 %). Gene products often participate in multiple processes, and a single gene can therefore be annotated to multiple GO terms. Of the transcripts with GO annotations, about 24 % (36,930) were assigned to just one GO term, more than 75 % (117,529) were assigned to over two GO terms, and about 3 % (5,563) can be assigned to over ten GO terms (Additional file 3). All these transcripts are important resources for genetic manipulations of *Miscanthus* in the future. Furthermore, the networks of gene interactions in cells could be well understood by the KEGG pathway analysis. In total, 11,405 transcripts matched 266 KEGG pathway annotations (KOs) were assigned into 32 KEGG pathway levels covering five main categories including organismal systems (1,770; 15.5 %), cellular processes (1,137; 10 %), environmental information processing (928; 8.1 %), genetic information processing (2,558; 22.4 %), and metabolism (4,860; 42.6 %) (Fig. 5). The three most represented pathway levels were “translation”, “signal transduction”, and “biosynthesis of other secondary metabolites”, followed by “folding, sorting and degradation” and “metabolism of terpenoids and polyketides”, whereas “sensory system” and “membrane transport” pathways represented the smallest categories. The most enriched pathways of these *Miscanthus* transcripts are given in Additional file 4, which mainly included ribosome (ko03010), carbon metabolism (ko01200), spliceosome (ko03040), biosynthesis of amino acids (ko01230), and Protein processing in endoplasmic reticulum (ko04141) pathways.

As *Miscanthus sinensis* is a potentially high-yielding bioenergy crop, cell wall biosynthesis/assembly is of fundamental importance to the biomass accumulation and utilization of the plant. Thus, a total of 1,108 transcripts associated directly or indirectly with cell wall biosynthesis and assembly were sorted out in Additional file 5 according to the annotation information of all transcripts (Additional file 2). Most of the identified transcripts were correlated with genes involved in the biosynthesis of three major cell wall components, including lignin, cellulose and hemicellulose. Lignin biosynthesis is a complex process that involves many enzymatic reactions. Almost all genes involved in each step of the monolignol biosynthetic pathway have been identified in the *Miscanthus* transcriptome, including *phenylalanine ammonialyase (PAL), 4-coumarate-CoA ligase* (*4CL*), *Cinnamate 4-hydroxylase* (*C4H*), *caffeoyl CoA 3-O-methyltransferase* (*CCoAOMT*), *Caffeic acid 3-O-methyltransferase* (*COMT*), *cinnamyl alcohol dehydrogenase* (*CAD*), *cinnamoyl-CoA reductase* (*CCR*), *hydroxycinnamoyl CoA shikimate hydroxycinnamoyl transferase* (*HCT*), *p-coumaroylshikimate 3′-hydroxylase* (*C3′H*), and *ferulic acid 5-hydroxylase* (*F5H*). Cellulose is mainly synthesized by members of cellulose synthase (CESA) and cellulase enzymes, which were representative ones found in the cellulose-related transcripts. *KORRIGAN* (*KOR*) and *COBRA* family genes that may be involved in the assembly of crystalline cellulose were also identified. We have also identified numerous hemicellulose biosynthesis related transcripts of *Xyloglucan endo-transglycosylase* (*XTH*), *Xyloglucan 6-xylosyltransferase* (*XXT*), *Xyloglucan glycosyltransferase* (*XGT*), *Xylosyltransferase* (*XT*) and *Glucan synthase* (*GS*) subfamily genes, indicating that Xyloglucans maybe the predominant hemicelluloses in the *Miscanthus* cell walls. In addition to the three major cell wall components, a minor proportion of cell wall structural protein related transcripts have also been found in the transcriptome, such as pectin related genes and cell wall loosening genes *expansin* (*EXP*). Additionally, transcripts of other *glycosyltransferases*(*GTs*)genes may also be related to cellulose or hemicellulose biosynthesis. The identification of genes involved in the formation of cell wall components and structures could provide insight into the molecular mechanisms underlying biosynthesis and assembly of cell wall in *Miscanthus* and thus serve as good candidates for future functional studies for improving the biomass properties of *Miscanthus*.

### Transcription factors

**Fig. 6:**
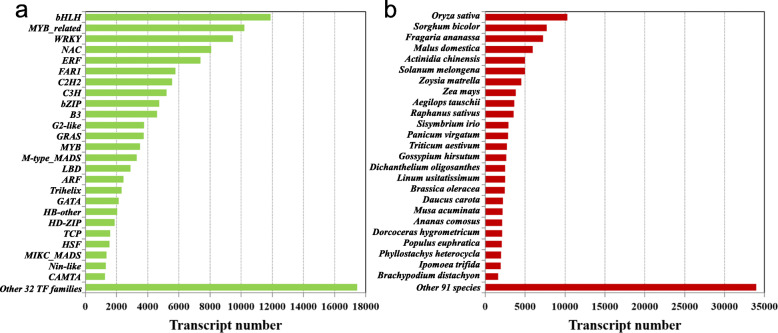
Classification of transcripts encoding transcription factors (TFs). **a** Distribution of the top 25 and other 32 TF families predicted by PlantTFDB. Bars represent the numbers of transcripts distributed to different TF families. **b** Species distribution diagram of TFs. Bars represent the numbers of transcripts distributed to different species

Transcription factors (TFs) play critical roles in various plant developmental processes by regulating transcription to switch genes on and off. They act either alone or in a coordinated fashion to trigger many fundamental genomic processes such as cell division, cell death, and development, and periodically react to signals coming from outside the cell [39]. The TF-encoding *Miscanthus* transcripts were annotated by comparing all the 408,801 integrated transcripts against the plant transcription factor database (PlantTFDB v3.0) [40]. A total of 125,608 (~ 30.7 %) TFs were identified to be broadly distributed against 116 plant species (Additional file 6). Most TF transcripts were annotated from the well characterized rice database, followed by sorghum, which showed very high genome mapping ratios to *Miscanthus* transcripts (Fig. 6a). These TF transcripts were from 57 annotated TF families, of which the top 25 identified families have been listed in Fig. 6b. The largest five families included bHLH (11,877; 9.5 %), MYB (10,219; 8.1 %), WRKY (9,479; 7.5 %), NAC (8,071; 6.4 %), and ERF (7,392; 5.9 %), which are all well known and most studied plant transcription factors. Additional noteworthy families with high numbers of transcripts included FAR1-like, C2H2, C3H, bZIP, and B3 families. The high prevalence of TFs identified in our transcriptome suggest relatively complex transcriptional regulation in *Miscanthus* plants.

### Identification of alternative splicing in *Miscanthus*

**Fig. 7:**
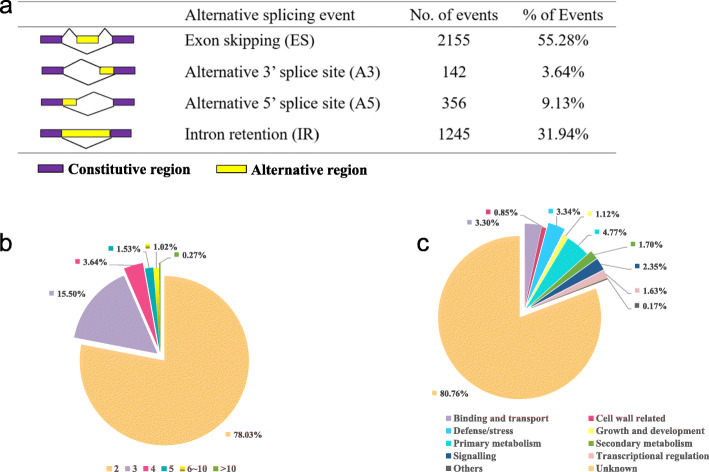
Alternative splicing events and splice isoform analysis with Iso-Seq isoforms. **a** Summary of the different types of alternative splicing events that generate alternative transcripts. **b** Pie chart showing the percentage of genes with different number of isoforms; different colours indicate genes with different number of isoforms. **c** Pie charts represent functional classification of the identified alternative spliced isoforms

Alternative splicing (AS) is a crucial regulatory mechanism that played an important role in understanding transcriptome and proteome diversity [41]. The extent and complexity of AS has been studied in several model plants with reference genomes mainly using high-throughput next-generation sequencing [14]. However, it is difficult to use this technique to examine AS in species without well-annotated reference genomes, especially in heterozygosity species. Although short-read sequencing transcriptome analysis of *Miscanthus* have previously been investigated, a precise prediction and identification of the alternative transcript splicing has not been possible in this potentially very complex transcriptome. However, the newest PacBio sequencing enables examining alternative splicing events without using a reference genome, 240,665 consensus full-length transcripts were used to identify alternative splicing events by using the *de novo* pipeline [23]. A total of 3,898 alternative splicing events were identified, including 2,155 (55.28 %) exon skipping events, 1,245 (31.94 %) intron retentions, 142 (3.64 %) alternative 3’splice sites, and 1,356 (9.13 %) alternative 5’splice sites (Fig. 7a). Among the AS genes, more than half of them (78.03 %) possess only two isoforms, 15.50 % genes possess three isoforms, 3.64 % genes possess four isoforms, 1.53 % genes possess five isoforms, 1.02 % genes possess more than six isoforms and 0.27 % genes have > 10 isoforms (Fig. 7b). Among them, 2,936 isoforms were annotated from the Nr database and sorted into 10 broad functional categories according to their predict functions (Fig. 7c, Additional file 7). The major category included isoforms whose functions have not yet been ascertained, which were assigned as ‘unknown’ category. The rest noticeably enriched categories were primary metabolism (4.77 %), defence/stress (3.34 %) and binding and transport (3.30 %). Besides, 0.85 % isoforms were assigned to ‘cell wall related’ category and 1.63 % isoforms were assigned to ‘transcriptional regulation’ category, these isoforms are worth further studies to reveal their regulatory functions on cell wall biosynthesis/assembly.

## Discussion

*Miscanthus* is one of the most promising C4 perennial non-food bioenergy grasses for cellulosic biofuel production. Functional genomic resources of *Miscanthus* are required to understand the molecular processes underlying their suitability for bioenergetics applications. With the development of new sequencing techniques, transcriptome analysis has been proven to be a very powerful means of gene discovery, genome annotation, and deep exploration of genes that contribute to phenotypic traits [6–10]. A few NGS RNA-Seq transcriptome databases of *M. sinensis*, *M. sacchariflorus*, and *M. lutarioriparius* have been reported previously [10, 11, 42], but these studies were limited by either transcript length and/or the number of transcript isoforms. Recently, the third-generation sequencing technology PacBio Iso-Seq opened up a new era of transcriptome-wide research, which is particularly suitable for the direct generation of comprehensive transcriptomes with accurate alternative splicing isoforms and novel genes in non-model organisms that lack genomic sequences.

In this study, we conducted a comprehensive transcriptomic analysis of *M. sinensis* using the PacBio Iso-Seq technique combined with the Illumina HiSeq platform. This hybrid approach provided a high-confidence transcriptomic atlas of *Miscanthus* that can serve as the genetic background necessary for basic biological research in *Miscanthus*. Approximately 6.6 Gb of Illumina HiSeq data and 15.7 Gb of PacBio Iso-Seq data were produced for each *M. sinensis* sample. The PacBio Iso-Seq data produced from the newest PacBio Sequel platform boast a much higher throughput than previous platforms were able to produce [16]. The ratio of full-length transcripts and mean length of transcripts were both greatly improved in our dataset (Fig. 1). The PacBio Iso-Seq data yielded an average of 590,062 high-quality FLNC reads with 240,665 (N50 of 2,863) consensus isoforms, which were of high-quality compared to previously detected maize full-length transcriptome data (258,948 FLNC reads, 111,151 transcripts with N50 of 2,632) and sorghum full-length transcriptome data (442,319 FLNC reads, 27,860 transcripts with N50 of 1,042) [18, 19].

The PacBio Iso-Seq results showed high-efficiency in recovering full-length transcripts. PacBio transcripts averaged 2.5 kb in length, while the average length of the Illumina transcripts was only 505 bp. The length of the PacBio Iso-Seq results appeared to more closely correspond to the true transcript length seen in plants (i.e. 1–3 kb). However, the principal weakness of the PacBio platform is that its sequencing depth is lower than that of Illumina. More unique transcripts were generated from the Illumina *de novo* assembly in this study than from the PacBio, which is in agreement with some other Illumina and PacBio Iso-Seq hybrid studies [24, 25]. This could be due to the high coverage of Illumina HiSeq technology as well as the fact that a large number of untrue transcripts tend to be unavoidably generated in the part of the Illumina *de novo* assembly process, which could also be the reason for the lower mapping ratios of the Illumina data than the PacBio data. On the other hand, the 189,406 common transcripts detected by the two datasets provided us with a lot of high-quality full-length transcripts for more accurate transcriptomic analysis of *Miscanthus*. Overall, our study highlights the strengths as well as limitations of PacBio data, which further highlighted the importance of integrating sequencing technologies to provide improved transcriptome analysis.

Compared with previous short-read sequencing studies of *Miscanthus*, the hybrid transcriptome sequencing performed in our study produced a more comprehensive transcriptome with several notable features. Firstly, more accurate full-length transcripts (with 408,801 completed open reading frames (ORFs)) were generated, which is almost fourfold the number of transcripts documented in previous *M. sinensis* (with 114,747 completed ORFs) and *M. lutarioriparius* (with 169,064 completed ORFs) transcriptome projects [10, 42]. The average transcripts length (1,685) in this study was also much longer than those in the previously published datasets for *M. sinensis* (1,288 bp) and *M. lutarioriparius* (759 bp). Thus, the high-quality full-length transcripts produced in our study can be used as reference to improve genome assembly and gene annotation of *M. sinensis*. Secondly, a total of 3,898 alternative splicing events of *M. sinensis* were identified for the first time, and these could provide a useful resource for investigating the potential functions of transcript isoforms of each gene to improve the accuracy of existing gene models. Thirdly, our dataset provides new evidence for a phylogenetic relationship of *M. sinensis* and sugarcane, while sorghum and maize represent more distant relatives. As such, our analyses contribute to a better understanding of *Miscanthus* evolution. Finally, our new transcriptome data not only provide additional valuable genomic resources for *Miscanthus*, but also provide data for comparing the efficiency of two sequencing methods, PacBio Iso-Seq and Illumina HiSeq, which can be used for further gene discovery and identification of molecular markers.

Functional annotation and classification of all transcripts provided rich information about the *Miscanthus* transcriptome. Using different databases for annotation can shed light on intracellular metabolic pathways and biological behaviors of *Miscanthus* genes. Approximately 75 % of non-redundant transcripts were annotated by sequence similarity search in public databases. The percentage of annotated transcripts was lower than those obtained from NGS data for *M. sinensis* (83.57 %) in a previous analysis [10], which is no surprise because we identified nearly fourfold transcripts than this previous study. It is also obvious that more novel unique *Miscanthus* transcripts were produced in our study by a combination of PacBio Iso-Seq with Illumina HiSeq technologies. Using GO and KEGG annotation, we have identified a large number of transcripts involved in metabolism, cellular processes, catalytic activity, binding, translation, and signal transduction, which were similar to previous studies in *M. sinensis*, *M. sacchariflorus*, and *M. lutarioriparius* [10, 11, 42], suggesting that our transcripts are representative of a comprehensive *Miscanthus* transcriptome.

Furthermore, our in-depth functional analysis of all transcripts led to the identification of a substantial number of genes involved in biosynthesis of cell wall components such as cellulose, hemicellulose and lignin, as well as pectin related genes and cell wall loosening genes involved in cell wall assembly and modification, indicating that cell wall biogenesis of *Miscanthus* is a very complicated process. The cell wall plays an essential role in determining cell size and shape, and thus positively contributes to biomass accumulation. Despite significant progress in cell wall biosynthesis in plant species with available genome resources, much less is known about the molecular mechanisms underlying cell wall biosynthesis, assembly and modification in the bioenergy crop *Miscanthus*. For example, studies on the down-regulation of lignin biosynthesis genes *COMT*, and *CAD* in switchgrass and maize showed a decrease in total lignin content and an increase in saccharification efficiency and ethanol production when compared with the non-transgenic plants [43–45]. While overexpression of *F5H* in transgenic poplar became more digestible and had improved pulping performance relative to control, which was attributed to the altered S/G ratio rather than the total lignin content [46]. Cellulose crystallinity is reportedly as another key parameter negatively affecting biomass digestibility. Assessments of some *CESA* gene mutants, such as *OsCESA9* and *FC17/CESA4* mutants of rice have been made to show an enhancement in biomass saccharifcation effciency, which could be caused by a significant reduction in cellulose crystallinity [47, 48]. Although effects of hemicellulose composition, pectin, and expansin on plant biomass recalcitrance have also been reported [49–51], the direct genetic manipulation of their genes to reduce recalcitrance has rarely been reported. Beside these cell wall related genes, a substantial number of TFs have also been proved to play substantial roles in the regulation of cell wall biosynthesis and assembly [52]. In our study, a wide variety of transcripts were predicted to be *Miscanthus* transcript factors, including more than 57 TF families (Additional file 6). Among them, a substantial number of *NAC* and *MYB* family genes were proved to play pivotal roles in the regulation of cell wall biosynthesis mostly in *Arabidopsis*, rice and maize [53, 54]. *NAC* factors act as the main switch in cell wall regulation, which orchestrate a large number of downstream *MYB* TFs and cell wall biosynthesis genes [55, 56]. *HD-ZIP* and *WRKY* family TFs have also been suggested to play important roles in cell wall formation [57, 58]. Our results thus contribute to the genome-wide study of cell wall related genes and regulators in *Miscanthus*. Further functional studies of these candidates will help develop novel strategies to genetically modify the cell walls of *Miscanthus* and improve its saccharification efficiency in bioethanol production.

Moreover, we detected a total of 3,898 alternative splicing events that were not previously identified in *Miscanthus* with high confidence. Alternative splicing plays important roles in regulating molecular, cellular, physiological, and developmental processes/pathways in eukaryotes. It has been reported that 95 % of intron-containing genes in humans and over 60 % of multi-exon genes in plants are alternatively spliced [59]. Most nuclear gene-related splicing events in plants have been found to involve different modes such as intron retention, alternative splicing, or exon skipping/inclusion [60]. It should be noted that the *de novo* pipeline we used to identify AS only has a 66–76 % overall success rate when used without a reference sequence [23]. Thus, only part of the alternative splicing events could be confirmed in our study in the absence of a reference genome Besides, it was found that the largest proportion of AS events identified in the *Miscanthus* transcriptome was exon skipping, which is not consistent with reports from other plants such as in maize [18], sorghum [19], and strawberry [25] where intron retention is the most common splicing mode. This could be distributed to the time and spatial features of transcriptome as well as the drawbacks to underestimate AS in the pipeline we used [23]. Nevertheless, our characterization of AS in the *Miscanthus* transcriptome provided a rich data resource for further functional studies of different isoforms. For example, the key C4 photosynthesis genes, such as *phosphoenolpyruvate carboxylase* (*PEPC*) and *pyruvate orthophosphate dikinase* (*PPDK*), were obviously identified in the ‘primary metabolism’ category with more than 10 alternative splicing isoforms (Additional file 7). *PEPC* and *PPDK* are two important enzymes that catalyse the early steps in the photosynthetic assimilation of CO_2_ in C4 plants [61]. The regulation of the *PEPC* and *PPDK* alternative spliced isoforms may be directly related to the photosynthesis efficiency of plants. Besides, cell wall related genes, especially lignin biosynthesis related genes, such as *4CL*, *CCoAOMT*, *COMT* and *CCR* genes were also identified to possess 2–4 alternative splicing isoforms in our transcriptome. Since photosynthesis and cell wall assembly are both critical for the biomass accumulation of *Miscanthus* plants, detailed studies of the alternative splicing isoforms of these genes are needed to reveal their regulatory mechanisms and genetic functions.

## Conclusions

Our study provides the first full-length functional transcriptome of *M. sinensis* derived from combined analyses of PacBio Iso-Seq and Illumina HiSeq datasets. The PacBio Iso-Seq analysis recovered more full-length transcripts with a longer N50, while the Illumina HiSeq analysis increased the whole transcript number and provided a greater sequencing depth. By taking advantage of the strong complementary nature of these two datasets, a total of 408,801 integrated transcripts were generated. The extremely high mapping ratios between the *Miscanthus* transcriptome and genome demonstrated the reliability of our transcriptome analysis. The close relationship of the *Miscanthus* transcriptome with the sugarcane and sorghum genomes indicated a high utility of sugarcane and sorghum as references for *Miscanthus*. In total, 306,228 (~ 75 %) transcripts were properly annotated to at least one database including NR, Swiss-Prot, eggNOG, GO, and KEGG. Numerous candidate genes involved in cell wall synthesis and modification were provided as good genetic materials to study and improve the *Miscanthus* biomass properties. Moreover, the predicted TF transcripts and identified alternative splicing events allowed us to pinpoint a wealth of candidate molecules mainly involved in the regulatory networks of *Miscanthus*. A major challenge for future will be to associate the different expression levels of splice isoforms with species-specific functional and phenotypic traits. Taken together, our transcriptome sequencing of *M. sinensis* provides useful information for gene identification and greatly enriches the available transcriptomic resources for *Miscanthus*, which will facilitate further advancements in deciphering the genetic and molecular mechanisms underlying the economically important traits of the species.

## Methods

### Plant material

Two genotypes of *Miscanthus sinensis*, B0605 and C0542, were selected from the *Miscanthus* germplasm nursery at the Hunan Agricultural University. Each line was propagated asexually via rhizomes to 12 individuals and grown in plastic pots with soil mixture (50 % loam, 50 % fine sand) and cultured in a greenhouse. After a 3-month establishment period, leaves and young stems were collected from four plants and pooled for PacBio Iso-Seq sequencing. The same tissues were collected from the other two sets of four plants and used as two biological replicates for Illumina HiSeq sequencing. Samples were snap-frozen in liquid nitrogen within 1 min after being excised and stored at − 80 °C until RNA extraction.

### RNA isolation and assessment of quality

The total mRNAs of each sample were isolated using Trizol reagent (Invitrogen) according to the manufacturer’s instructions. Genomic DNA was then removed by DNase I (Promega). To ensure the accuracy of sequencing data, the quality of mRNAs was initially evaluated by electrophoresis in 1.5 % agar gel, and then quantified using the NanoDrop2000 spectrophotometer (ThermoFisher Scientific) and Agilent 2100 Bioanalyzer (Agilent Technologies). Only samples with the RNA integrity number (RIN) values higher than 8.0 were subjected for further analysis.

### PacBio Iso-Seq library preparation and sequencing

Library construction and PacBio sequencing were performed according to the official protocol described by Pacific Biosciences (Pacific Biosciences, Menlo Park, CA, USA). Briefly, total mRNAs (~ 15 µg) of each sample was reversely transcribed into cDNA using the SMARTer™ PCR cDNA Synthesis Kit (Clontech, CA, USA) that was optimized for preparing high-quality, full-length cDNAs. The amplified cDNA products were purified for library construction using the SMRTbell template prep kit 1.0. Libraries by annealing a sequencing primer and adding polymerase to the primer-annealed template. The polymerase-bound template was bound to MagBeads and SMRT sequencing was then performed on the Pacific Bioscience Sequel System using the Sequel Sequencing kit 2.1. Sequence movie files from the two datasets were processed and analyzed through the Iso-Seq pipeline using PacBio SMRT Analysis Server v2.3.0 (http://www.pacb.com/products-andservices/ analytical-software/smrt-analysis/) to filter out polymerase reads < 50 bp and quality < 0.8 with 0 minimum full passes (Table 1). This allows for the highest yield of reads of insert (ROIs) consensus sequences going into the subsequent steps, while creating higher accuracy consensus sequences where possible. The filtered ROIs were classified into four categories: full-length non-chimeric (FLNC), full-length chimeric, non-full-length, and short reads. This is done by identifying the 50 and 30 adapters used in library preparation as well as the poly (A) tail. Only reads that contain all three in the expected arrangement and do not contain any additional copies of the adapter sequence within the DNA fragment are classified as full-length non-chimeric. Furthermore, FLNC ROIs sequences were passed through the isoform-level clustering algorithm ICE (Iterative Clustering for Error Correction). ROI sequences were used to correct errors (polish) in the isoform sequences using the Quiver software module. The polishing process of Quiver generated high-quality (HQ) isoform sequences to an expected accuracy of ≥ 99 %. Finally, the high-quality consensus transcripts of libraries from the two samples were merged and redundancy was removed using CD-HIT-EST (c = 0.95) to obtain final FL isoforms for further analysis [62].

### Illumina HiSeq sequencing and *de novo* assembly

Total mRNAs were extracted from each sample and evaluated as described above. A total of about l0 ug mRNAs per sample was used as input material to generate sequencing libraries using the NEBNext Ultra™ RNA Library Prep Kit for Illumina (NEB) following the manufacturer’s recommendations, and index codes were added to attribute sequences to a specific sample. Briefly, mRNAs were purified using poly-T oligo-attached magnetic beads. The first strand cDNAs were synthesized from the total mRNAs with random hexamer primers, followed by second strand cDNAs synthesis using DNA polymerase I (New England BioLabs) and RNase H (Invitrogen). After end repair, adaptor ligation, and index codes adding for each sample, PCR amplification was conducted. Four cDNA libraries were synthesized and sequenced respectively on the Illumina HiSeq 2500 platform to obtain 150 bp paired-end read data. Raw data (raw reads) in fastq format were first processed using Cutadapt (v1.15). In this step, clean data (clean reads) were obtained by removing reads containing adapters, reads containing poly-N and low-quality reads. These clean reads were then mapped to PacBio isoform sequences using Bowtie 2 (ver. 2.26) [26]. For the unmapped reads, *de novo* assembly was performed using the Trinity program with K-mer size of 25 to produce complementary transcripts [27].

### Transcriptome combination and mapping

Both full-length transcripts generated by PacBio- and Illumina-derived datasets from different *Miscanthus sinensis* lines were merged into a final dataset, which was further clustered by CD-HIT-EST ( c = 0.95) to remove redundancy and produce the final transcripts for subsequent analyses. To assess the completeness of the conserved content of the final transcripts, the BUSCO v10 dataset was used in BUSCO (Benchmarking Universal Single-Copy Orthologs) analysis [28]. The percentages of the conserved proteins that fully and partially aligned to transcripts were calculated. To assess the transcriptome completeness and genetic relationships with several closely related species, each library and the final transcripts were aligned individually against the genomes of miscanthus (Msinensis_497_v7.0.fa.gz), rice (Oryza_sativa.IRGSP-1.0.dna.toplevel.fa.gz), maize (Zea_mays.B73_RefGen_v4.dna.toplevel.fa.gz), sorghum (Sorghum_bicolor.Sorghum_bicolor_NCBIv3.dna.toplevel.fa.g), and sugarcane (GCA_003544955.1_Sspon.HiC_chr_asm_genomic.fna.gz). TopHat (v2.0.14) was used for the mapping of Illumina libraries with a tolerance of 4 mismatches per read and the rest set as default [63]. GMAP was used for the mapping of PacBio libraries and the final transcripts with 80 % identity and 90 % coverage threshold and the rest set as default [64].

### Functional annotation of transcripts

Functional annotations for all transcripts were performed by BLAST similarity search against NCBI NR (NCBI non-redundant protein sequences), Swiss-Prot (a manually annotated and reviewed protein sequence database) and eggNOG (evolutionary genealogy of genes: Non-supervised Orthologous Groups) databases (E-value: 10^− 5^) to obtain the NR, Swiss-Prot and eggNOG annotations. Homology searches were carried out by query of the NCBI non-redundant protein database by using BLASTx (E-value, 10^− 5^). Gene names were assigned to each assembled sequence based on the best BLAST hit. The BLAST results were then imported into the Blast2GO program (https://www.blast2go.com/) to map the sequences into GO terms. WEGO software was used to analyze the GO functional classification for the transcripts. KEGG pathway analysis was performed using the KEGG Automatic Annotation Server (KAAS) (http://www.genome.jp/tools/kaas/). The TF-encoding transcripts were annotated by comparing the CDS sequences of the final transcripts against the Plant Transcription Factor Database (PlantTFDB v3.0, http://planttfdb.gao-lab.org/prediction.php) [40] with default built-in settings.

### *De novo* detection of alternative splicing events

To further detect alternative splicing events involved in the current *Miscanthus sinensis* transcriptome, a *de novo* pipeline by clustering transcript sequences from the same gene and determining if insertion appeared in the alignment were used in this study. The pipeline has already been successfully applied to *Amborella trichopoda* and *Brassica napus* with more than 66 % accuracy [23, 65]. First, high-quality PacBio FLNC reads were clustered by CD-HIT-EST (c = 0.99) to filter sequences with similarity rates over 99 % and length difference less than 1 %. Based on the *de novo* mapping strategy, the all-vs-all BLAST with high identity settings was then run on the cluster.fasta got from the first step. Finally, four major types of AS events, namely IR (intron retention), ES (exon skipping), A3 (alternative 3’splice sites), and A5 (alternative 5’splice sites), were extracted from the output files and counted.

## Supplementary Information

**Additional file 1.** Transcriptome coverage analysis based on the BUSCO alignment.

**Additional file 2.** Transcript annotation of *Miscanthus sinensis* based on public databases. NA indicates no information found.

**Additional file 3. **Overlap analysis of GO terms of *Miscanthu1s* transcripts. Pie charts represent classification of the number of GO terms of each transcripts.

**Additional file 4.** KEGG pathways enriched of transcripts.

**Additional file 5.** Identification of the cell wall related genes and their representative transcripts.

**Additional file 6.** Annotated information of transcription factors based on the PlantTFDB v3.0 database.

**Additional file 7.** Annotated information of the alternative spliced isoforms based on the NR database.

## Data Availability

The data generated in this study, including PacBio Iso-Seq reads and Illumina short reads, have been submitted to the Sequence Read Archive (SRA) of the National Center for Biotechnology Information (NCBI) under accession numbers SRP235391 and SRP235673. The assembled transcripts including PacBio isoforms and Illumina unigenes have been deposited in the NCBI TSA under the accession numbers GJAM00000000 and GJAL00000000.

## Abbreviations

AS: Alternative splicing
COG/KOG: Cluster of orthologous groups of proteins
eggNOG: Evolutionary genealogy of genes:Non-supervised Orthologous Groups
GO: Gene Ontology
KAAS: KEGG Automatic Annotation Server
KEGG: Kyoto Encyclopedia of Genes and Genome
NGS: Next-generation sequencing
Nr: NCBI non-redundant protein sequences
ORFs: Open reading frames
ROIs: Reads of inserts
Swiss-Prot: A manually annotated, non-redundant protein database
TAPIS: Transcriptome analysis pipeline for isoform sequencing
TFs: Transcription factors
