# Supplementary figures and images for: Unveiling the transcriptomic complexity of Miscanthus sinensis using a combination of PacBio long read- and Illumina short read sequencing platforms
Source: BMC Genomics. 2021 Sep 22;22:690. doi: 10.1186/s12864-021-07971-x (PMC8459517; doi:10.1186/s12864-021-07971-x)

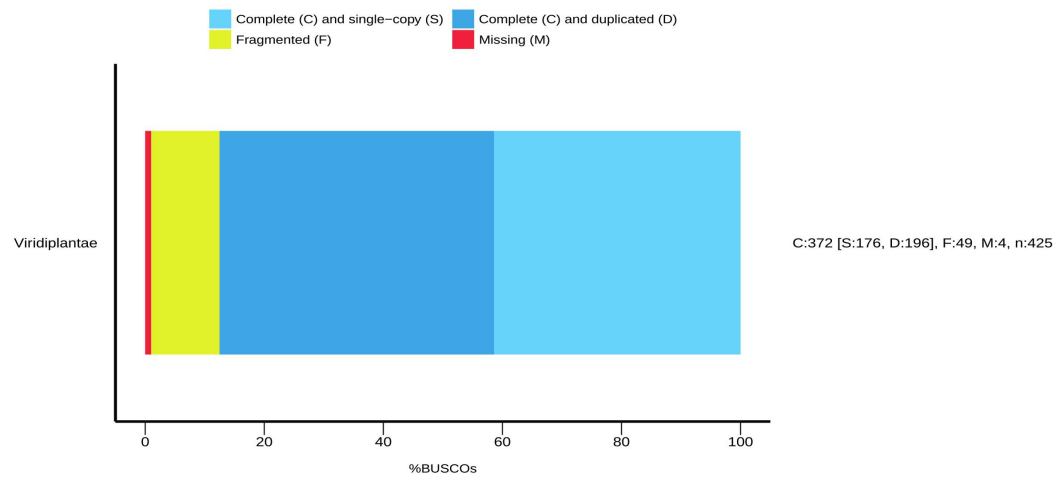

Supplement: Supplementary file 1 — Additional file 1. Transcriptome coverage analysis based on the BUSCO alignment. [file 12864_2021_7971_MOESM1_ESM.pdf]

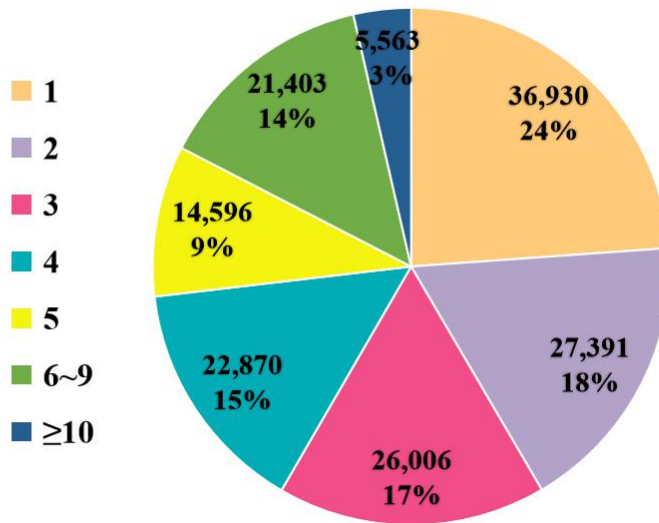

Supplement: Supplementary file 3 — Additional file 3. Overlap analysis of GO terms of Miscanthu1s transcripts. Pie charts represent classification of the number of GO terms of each transcripts. [file 12864_2021_7971_MOESM3_ESM.pdf]
